# Highly Efficient Computationally Derived Novel Metagenome α-Amylase With Robust Stability Under Extreme Denaturing Conditions

**DOI:** 10.3389/fmicb.2021.713125

**Published:** 2021-08-30

**Authors:** Shohreh Ariaeenejad, Behrouz Zolfaghari, Seyedeh Fatemeh Sadeghian Motahar, Kaveh Kavousi, Morteza Maleki, Swapnoneel Roy, Ghasem Hosseini Salekdeh

**Affiliations:** ^1^Department of Systems and Synthetic Biology, Agricultural Biotechnology Research Institute of Iran, Agricultural Research Education and Extension Organization, Karaj, Iran; ^2^Department of Computer Science and Engineering, Indian Institute of Technology Guwahati, Guwahati, India; ^3^Laboratory of Complex Biological Systems and Bioinformatics, Department of Bioinformatics, Institute of Biochemistry and Biophysics, University of Tehran, Tehran, Iran; ^4^School of Computing, University of North Florida, Jacksonville, FL, United States; ^5^Department of Molecular Sciences, Macquarie University, Sydney, NSW, Australia

**Keywords:** α-amylase, metagenome, stability, *in silico* screening, detergent-compatible

## Abstract

α-Amylases are among the very critical enzymes used for different industrial purposes. Most α-amylases cannot accomplish the requirement of industrial conditions and easily lose their activity in harsh environments. In this study, a novel α-amylase named PersiAmy1 has been identified through the multistage *in silico* screening pipeline from the rumen metagenomic data. The long-term storage of PersiAmy1 in low and high temperatures demonstrated 82.13 and 71.01% activities after 36 days of incubation at 4 and 50°C, respectively. The stable α-amylase retained 61.09% of its activity after 180 min of incubation at 90°C and was highly stable in a broad pH range, showing 60.48 and 86.05% activities at pH 4.0 and pH 9.0 after 180 min of incubation, respectively. Also, the enzyme could resist the high-salinity condition and demonstrated 88.81% activity in the presence of 5 M NaCl. PersiAmy1 showed more than 74% activity in the presence of various metal ions. The addition of the detergents, surfactants, and organic solvents did not affect the α-amylase activity considerably. Substrate spectrum analysis showed that PersiAmy1 could act on a wide array of substrates. PersiAmy1 showed high stability in inhibitors and superb activity in downstream conditions, thus useful in detergent and baking industries. Investigating the applicability in detergent formulation, PersiAmy1 showed more than 69% activity after incubation with commercial detergents at different temperatures (30–50°C) and retained more than 56% activity after incubation with commercial detergents for 3 h at 10°C. Furthermore, the results of the wash performance analysis exhibited a good stain removal at 10°C. The power of PersiAmy1 in the bread industry revealed soft, chewable crumbs with improved volume and porosity compared with control. This study highlights the intense power of robust novel PersiAmy1 as a functional bio-additive in many industrial applications.

## Introduction

Enzymes are one of the essential biological molecules with numerous benefits in various industries. These biocatalysts play a key role in many industry sectors due to their potential in cost-effective industrial processes reducing energy consumption, and diminishing environmental problems (Al-ghanayem, 2020). The harsh conditions such as extreme temperature and pH, heavy metals, toxic solvents, and salinity limit the application of enzymes. Therefore, the production of enzymes with stability over the wide range of pH and temperature, long-term storage, and other abnormal situations is crucial for industrial applications.

α-Amylases are one of the most common enzymes with a vital role in most industrial applications. These endo-acting enzymes attack the α-(1,4) glucosidic bonds in the starch and oligosaccharide substrates to liberate glucose, maltose, maltotriose, and low-molecular-weight dextrins (Mehta and Satyanarayana, 2016). α-Amylases are used in a wide array of industries related to starch processing, including detergent, textile, paper, fermentation, and food applications (Sindhu et al., 2017). The stability of the α-amylase under extreme conditions is an essential property in starch-based applications (Dai et al., 2020). To improve enzymatic stability and activity, several strategies can be used such as enzyme immobilization, enzyme cocktail development, and compatible solutes or osmolytes (Maleki et al., 2020; Norouzi et al., 2020; Ariaeenejad et al., 2021b).

One of the effective ways for improving enzyme function under extreme industrial conditions is to use powerful tools for identifying robust enzymes. α-Amylases can be derived from plants, animals, and microorganisms such as bacteria, fungi, yeasts, and archaeons (Gupta et al., 2003). Several cultivation approaches have been used to explore the diversity and microbial communities. However, these methods are biased due to the inaccessibility of more than 99% of the microorganisms in these approaches (Ahmad et al., 2019). The metagenomic technology has evolved to decrease the high cost of enzyme production and paved the way for the identification of novel enzyme from the uncultivable microbial communities (Ahmad et al., 2019). Through the metagenomics approach, several α-amylases were identified to meet the industrial application (Sindhu et al., 2017). A novel halotolerant α-amylase was identified from the metagenomic library of marine sediment (Nair et al., 2017). Another study used the sea metagenomic data to produce a novel α-amylase with particular textile and food industries (Nair and Bhat, 2020). Using the metagenomic library of cow dung, a novel α-amylase has been identified for potential application in industries (Pooja et al., 2015). In other studies, novel thermostable α-amylases have been identified from the rumen metagenomic data to be used in food and poultry industries (Motahar et al., 2020; Sadeghian Motahar et al., 2021). Even though metagenomics gives us enormous wealth, choosing the best enzyme for its industrial use is still challenging. Using the multistage *in silico* screening method through computational guidance instead of the simple alignment-based screening demonstrated the high efficiency in producing novel enzymes with superior stability under harsh conditions (Ariaeenejad et al., 2020a, 2021a). Moreover, applying this pipeline and using the rumen metagenomic data resulted in the discovery of stable enzymes with high activity in several harsh conditions for industrial usage (Ariaeenejad et al., 2020b, c). The robust, stable α-amylases with a broad spectrum of stability and specificity have found applications in many industrial processes due to their ability to withstand harsh industrial conditions.

The cold-active alkaline α-amylases are a potential candidate for the detergent industry. These enzymes catalyze the reactions at low temperatures, reduce the release of harmful components to the environment, and enhance the ability to removing the harsh stains and improve the cloth softness, whiteness, and color (Al-ghanayem, 2020). Numerous examples can be cited from the literature indicating the high potential of alkaline, detergent-compatible α-amylases as an environmentally friendly component in stain removal and brightness of fabric (Hmidet et al., 2019; Allala et al., 2020; Kherouf et al., 2021).

α-Amylases have proven to be an excellent alternative catalyst in the food industry. The thermostable α-amylases were added to the bread formulation to enhance the quality of the final product (Wang et al., 2020). These starch-degrading enzymes increase the production of reducing sugars by yeasts responsible for improving the texture and volume of bread, enhancing the Maillard reactions, followed by the browning of the crust, and improving the flavor, taste, and color of the bread crumbs (Goesaert et al., 2009). Apart from these effects, the α-amylases can decrease the hardness of bread, produce a softer texture, retain the shelf-life, and act as anti-staling agents in baked products (Amigo et al., 2021). The addition of the novel thermostable α-amylase in bread formulation improved the textural and rheological properties of the wheat bread (Wang et al., 2020). Moreover, the cold-active α-amylases showed their effectiveness in baking processes by lowering the time needed for dough fermentation and keeping the aromas and moisture content of bread (Kuddus, 2018).

Given the urgent demand for sustainable applications, alkaline, cold-adapted thermostable α-amylases are the best alternative. Therefore, the current study aimed to use the metagenomic data of sheep rumen to introduce a novel highly stable α-amylase, PersiAmy1. The enzyme was an alkaline, stable α-amylase with a broad window of temperature and was strongly active under harsh conditions. PersiAmy1 can be used in many different industrial applications and showed high potential in the detergent and food industries.

## Materials and Methods

### Materials

3,5-Dinitrosalicylic acid (DNS), amylose, amylopectin, glycogen and pullulan, metal ions, Triton X-100, Tween 20, phenylmethylsulfonyl fluoride (PMSF), sodium dodecyl sulfate (SDS), ethylenediaminetetraacetic acid (EDTA), cetrimonium bromide (CTAB), dithiothreitol (DTT), urea, ethanol, methanol, wheat, potato, and corn starch were all from Sigma-Aldrich (Germany). The tested commercial detergents were from Persil (Henkel, Germany), and Softlan, Taj, Homecare, and Homecare base (Iran) were purchased from a local market (Karaj, Iran). The Luria-Bertani medium (LB broth), T4 DNA ligase (Thermo Fisher Scientific), kanamycin (Duchefa), isopropyl β-D-1-thiogalactopyranoside (IPTG), *Nde*I and *BamH*l restriction enzyme (Thermo Fisher Scientific), Gel Extraction Kit (Thermo Fisher Scientific, United States), and Ni-NTA Fast Start Kit (Qiagen, Hilden, Germany) were used for the cloning, expression, and purification of the enzyme (PersiAmy1) in the Agricultural Biotechnology Research Institute of Iran (ABRII).

### Bioinformatics Analysis of Screening Novel Robust Stable α-Amylase

After quality control and short-read assembly on sheep rumen metagenomics data, the FastQC and MEGAHIT assembler was used to evaluate raw metagenomic sequences’ quality. For the prediction of potential microbial genes, MetaGeneMark was employed. For the next step, the predicted α-amylases were separated from assembled contigs.

Some experimentally validated alkaline or/and cold-stable α-amylase sequences were selected by literature mining (Supplementary Table 1) and, using standalone NCBI BLAST, were aligned against enzymes mined from the metagenome. The predicted metagenomic α-amylase with the appropriate *E*-value was selected for further analyses. The existence of the α-amylase domain in predicted genes was confirmed by the NCBI Conserved Domains Database (CDD) (Marchler-Bauer et al., 2017). To validate the tertiary structure of selected metagenomic enzymes, their 3D models were predicted by the Phyre2 server (Kelley et al., 2015).

To determine the evolutionary position of enzymes extracted from the rumen metagenome, named PersiAmy1, the phylogenetic tree consisting of these enzymes and the 10 abovementioned known enzymes were inferred using the Neighbor–Joining method (Saitou and Nei, 1987).

The tree is drawn to scale for inferring the phylogenetic tree, with branch lengths in the same units as those of the evolutionary distances used. The evolutionary distances were computed using the number of differences method (Nei and Kumar, 2000) and are in the units of the number of amino acid differences per sequence. There were a total of 400 positions in the final dataset. Evolutionary analyses were conducted in MEGA X (Kumar et al., 2018). Eventually, by removing irrelevant or less relevant enzymes using mentioned stepwise filters, one of the sequences that passed all filters, named PersiAmy1, was selected for further analyses.

### Expression and Purification of the PersiAmy1

PersiAmy1 encoding sequence was amplified by polymerase chain reaction (PCR) using F (5′-TAATAGCATATG ATGAAA AAGTACCTCTTACC-3′) and R (5′-TGATAGGGATCC TTATTTGACGATGACGTATTC-3′) primers which included *Nde*I and *BamH*l restriction sites, respectively, and pET28a digested. Sequence data of PersiAmy1 with accession number MT560082 are shown in Supplementary Figure 1. The recombinant plasmid PersiAmy1-pet28a was transformed into *Escherichia coli* strain BL2 (DE3). The recombinant strain BL21/pET28a-PersiAmy1 was cultivated at 37°C in LB medium containing 50 μg/ml kanamycin; afterward, the expression of PersiAmy1 was induced by adding IPTG.

Eventually, induced cells were harvested by centrifugation and N-terminal Histidine-tagged recombinant protein was purified by utilizing Ni-NTA Fast Start Kit (Qiagen, Hilden, Germany). Fractions were evaluated by sodium dodecyl sulfate-polyacrylamide gel electrophoresis (SDS-PAGE).

### Zymogram Analysis

The zymogram analysis of PersiAmy1 was performed based on the previous report (Andrades and Contreras, 2017). In this regard, the SDS-PAGE gel containing 1% starch was performed under a non-reduced condition. The iodine solution was used to stain the gel and enzyme activity recognized by the clear zone on a dark-blue background.

### Enzyme Standard Assays

The activity of α-amylase was assayed according to the DNS procedure (Miller, 1959). In brief, the reaction mixture containing soluble starch (1% w/v) in Tris–HCl buffer (pH 9.0, 50 mM) and the enzyme (1 mg.ml^–1^) was incubated at 10°C for 20 min. Then, the DNS solution was added to the reaction mixture to stop the reaction and boiled for 5 min. The absorbance was read at 540 nm using a UV/visible spectrophotometer. One unit of α-amylase activity was defined as the amount of enzyme required for the liberation of 1 μmol reducing sugar per minute, and the specific activity of the enzyme was defined as unit per milligram of protein. The standard curve constructed by glucose and the protein content of the α-amylase were estimated using the Bradford method (Bradford, 1976).

### Influence of Temperature, and pH of the Reaction on α-Amylase Activity and Stability

The optimal pH of PersiAmy1 was examined by incubation of the purified enzyme with the starch (1% w/v) prepared in 50 mM carbonate-bicarbonate buffer (pH 4.0–5.0), phosphate buffer (pH 6.0–8.0), and Tris–HCl buffer (pH 9.0–11.0) at 10°C. The pH stability was analyzed by incubating the enzyme in the carbonate–bicarbonate buffer (pH 4.0–5.0), phosphate buffer (pH 6.0–8.0), and Tris–HCl buffer (pH 9.0–11.0) for 180 min. The enzymatic activity was measured after 30, 60, 120, and 180 min by DNS assay.

To explore the effect of temperature on PersiAmy1 activity, the enzyme was incubated with the starch (1% w/v) prepared in 50 mM Tris–HCl buffer (pH 9.0) at various temperatures extending from 0 to 100°C. For the thermal stability investigation, PersiAmy1 was incubated at different temperatures ranging from 50 to 90°C and the enzymatic activity measured after 30, 60, 120, and 180 min by the DNS method. The relative activity was measured by defining the control sample (0 h incubation) as 100% activity.

### Substrate Spectrum of the Purified PersiAmy1

To perform the substrate spectrum of PersiAmy1, different substrates including wheat, potato and corn starch, amylose, amylopectin, glycogen, and pullulan at 1% w/v concentration were used. The enzyme assay was conducted with each substrate. The activities of the samples were determined using the DNS method, and the relative and specific activities were calculated.

### Kinetic Studies of Enzyme Reaction

The kinetic parameters of PersiAmy1 were evaluated by preparing the starch in different concentrations (0.1–1 mg.ml^–1^) and measuring the activity according to the standard enzyme assay. The kinetic parameters including K_m_ and V_max_ were calculated according to the Lineweaver–Burk plot.

### Storage Stability of PersiAmy1

The storage stability of PersiAmy1 was analyzed by incubating the purified enzyme at 4 and 50°C for 36 days in 50 mM Tris–HCl buffer (pH 9.0). The amylase activity was measured in 4-day intervals using the DNS assay. The relative activity was calculated respecting to the maximum activity (0 h incubation) as 100%.

### Evaluation of PersiAmy1 Stability in the Presence of Metal Ions and Inhibitors

The activity of PersiAmy1 was estimated in the presence of different metal ions including Na^+^, K^+^, Ni^2+^, Cd^2+^, Mg^2+^, Ca^2+^, Mn^2+^, Fe^2+^, Co^2+^, Cu^2+^, Zn^2+^, and Pb^2+^ with a final concentration of 10 mM after 30 min of preincubation at room temperature.

To analyze the halotolerance of PersiAmy1, different concentrations of NaCl (0.05, 0.1, 0.5, 1, 2, 3, 4, and 5 M) were incubated with the enzyme for 30 min at room temperature. The enzymatic activity was measured via DNS assay.

The effect of several inhibitors and surfactants on the activity of PersiAmy1 was investigated in the presence of Triton X-100, Tween 20, SDS, PMSF, EDTA, CTAB, Urea, DTT, ethanol, and methanol in 10 mM concentration. The enzyme was preincubated with each additive for 30 min at room temperature. The amylase activity was determined under standard conditions.

For the mentioned experiments, the relative activities were measured under enzyme assay conditions and the activity in the absence of any additives was defined as control (100%).

### Potential Application of the Robust Stable PersiAmy1 in the Detergent Industry

#### Compatibility With Commercial Detergents

The compatibility and stability of PersiAmy1 against commercial laundry detergents were studied to confirm its potential as a suitable detergent additive (Priyadarshini and Ray, 2019). The tested detergents were Persil, Softlan, Taj, and Homecare. To inactivate any enzymes of detergents, they dissolved separately in the water to the concentration of 7 mg.ml^–1^ and heated at 90°C for 30 min. PersiAmy1 was added to the detergent solutions and incubated for different periods of time (0.5, 1, 2, and 3 h) at 10°C and various temperatures (30, 40, and 50°C) for 1 h. The remaining activities were estimated under standard assay conditions and samples without detergents used as control.

#### Wash Performance Analysis

The wash performance analysis of PersiAmy1 was performed by determining the chocolate stain releasing capacity of alkaline α-amylase according to the method of previous studies with some modification (Hammami et al., 2018). For this purpose, chocolate was heated at 70°C and 100 μl of the liquefied chocolate was stained to cotton fabrics (2.5 cm × 2.5 cm). Then the fabrics were dried overnight under a hot air oven. Each piece of clothes was washed under the following conditions: (a) 5 ml of tap water (control), (b) 4 ml of tap water and 1 ml of purified α-amylase, (c) 4 ml of tap water and 1 ml of heated detergent (7 mg.mL^–1^), and (d) 4 ml of tap water and 1 ml of heated detergent (7 mg.ml^–1^) containing purified α-amylase preparation. The untreated chocolate-stained cloth piece was considered as a control. The ability of PersiAmy1 to removing the stain examined visually.

### Potential Application of the Robust Stable PersiAmy1 in the Bakery Industry

#### Preparation of Dough and Bread

The wheat bread samples were prepared based on 100 g of total flour. For this goal, all dry ingredients (100% wheat flour, compressed yeast 3%, salt 2%) and liquid ingredients (sunflower oil 6% and water 80%) were mixed and blended thoroughly for 10 min at low speed until dough formation was complete. The enzyme was added to the dough mixture (0.008 U/g flour) and the batter was then fermented for 1 h at 40°C, and it was baked in a preheated oven at 150°C for 45 min. The bread was cooled at room temperature and kept in polyethylene packages before analyses and the sample without enzyme used as control.

#### Texture Profile Analysis

To perform the texture profile analysis (TPA) of bread samples 1 day after baking, they were sliced and their crusts were removed. The CT3 model Texture analyzer (Brookfield, United States) equipped with 10 kg load cell and a 12.7-mm aluminum cylindrical probe were used for the experiment. The test speed was 2 mm/s under compression of 40%. Textural properties of bread containing hardness, springiness, cohesiveness, and chewiness were measured. Three replications were performed for each bread type.

#### Color Analysis

The colors of bread were analyzed the day after baking by digital image analysis. For this goal, three parts of the crust were cut and images were taken using the HP ScanJet scanner. Pictures were tested using Fiji ImageJ software (version 1.52a). Ten points of images were chosen, and L^∗^ (brightness; 0: black, 100: white), a^∗^ (+a, redness; −a, greenness), and b^∗^(+b, yellowness; −b, blueness) values were recorded and their average measured. Browning index (BI) was calculated as follows (Bal et al., 2011):

(1) $$X=\left(a^{*}+1.75L^{*}\right)/\left(5.645L^{*}+a^{*}-3.012b^{*}\right)$$

(2) BI=[100(X-0.31)]/(0.17)

where BI is the browning index, L^∗^ is the brightness, a^∗^ is the redness, and b^∗^ is the yellowness.

#### Scanning Electron Microscopy

Small pieces of crumbs in 1-cm^3^ dimensions were cut from the center of samples and lyophilized for 24 h. The bread structure was examined using a scanning electron microscope (FEI ESEM Quanta 200, United States) at 15 kV.

#### Statistical Analyses

Results were analyzed using one-way analyses of variance (ANOVA), and the differences were tested on the 0.95 level. SPSS software version 22 was used to performed analyses. Three replications were conducted for experiments.

## Results

### Identification of PersiAmy1 as a Novel Robust Stable Amylase

**FIGURE 1:**
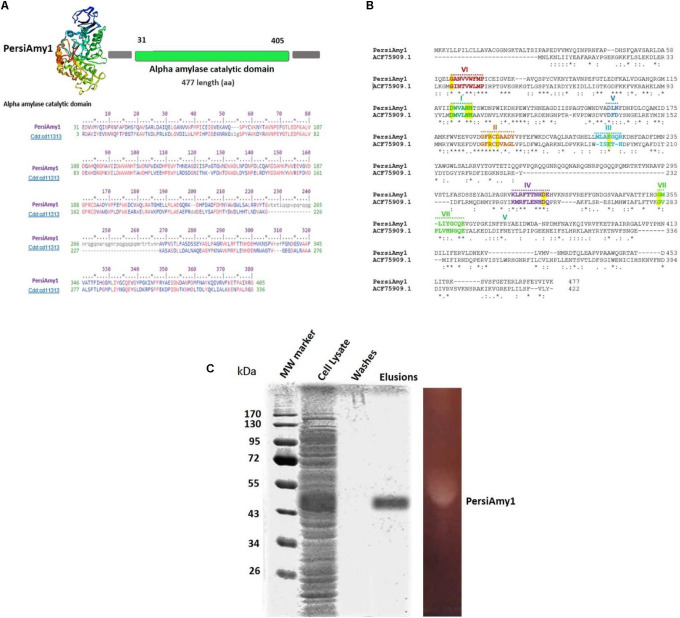
**(A)** Predicted 3D structure of PersiAmy1 obtained from the Phyre2 prediction server. The Conserved Domains Database (CDD) revealed the active sites and alignment of PersiAmy1 and the pfam00150 and cd14256 catalytic domain. **(B)** Comparison sequences of PersiAmy1 and *Thermotoga neapolitana* α-amylases (ACF75909.1) were done. The highly conserved sequences and regions are emphasized by a highlight. **(C)** Sodium dodecyl sulfate-polyacrylamide gel electrophoresis (SDS-PAGE) and zymogram analysis of PersiAmy1.

To identify an alkaline or/and cold stable α-amylase, the sheep rumen metagenome data was explored, and one of the assembled contigs, named PersiAmy1, passed all filters (Ariaeenejad et al., 2020b). The candidate α-amylase is an enzyme with the amino acid length of 477. The blast results show that it is most similar to a α-amylase (EC 3.2.1.1) from archaeal and bacterial α-amylases (also called 1,4-alpha-D-glucan-4-glucanohydrolase). PersiAmy1 best matches the Pssm-ID 200452 with Bit Score 420.42 and *E*-value 5.19e-146 based on the CDD results (Sadeghian Motahar et al., 2021). According to Phyre2 suggestion, the most similar structure to PersiAmy1 with 100% confidence and 21% identity belongs to an α-amylase from *Malbranchea cinnamomea* with 3VM7 PDB code (Figure 1A).

The evidence obtained from the comparative sequence alignment between PersiAmy1 with the characterized GH13 α-amylase, and existence of the conserved sequence region, suggests that PersiAmy1 had the closest sequence to *Thermotoga neapolitana* α-amylases (ACF75909.1) (Motahar et al., 2020).

Well-known conserved regions (I, II, III, IV, V, VI, and VII) (Cha et al., 1998; Park et al., 2010) and invariant catalytic residues of GH13 were evident in this comparison (Figure 1B). The phylogenetic tree constructed using 17 α-amylases with alkaline or/and cold-stable properties besides PersiAmy1 is demonstrated in Supplementary Figure 2.

### Expression and Purification of PersiAmy1

Under the control of the T7 promoter of the pET-28a vector in *E. coli* BL21 (DE3), the sequence of PersiAmy1 fragments was directly amplified with degenerate primers from the metagenomic DNA sheep rumen and overexpressed. The recombinant enzyme was purified as the N-terminus His-tagged protein purified using Ni-NTA Fast Start Kit, and fractions of the purification steps were analyzed by SDS-PAGE. According to Figure 1C, a single protein band was visible after the final purification step with a molecular weight of 53.70 kDa. Also, the zymogram analysis showed one clear zone, which confirmed the activity of PersiAmy1 by hydrolyzing starch as substrate.

### Influence of pH and Temperature on the Amylolytic Activity and Stability of the Enzyme

**FIGURE 2:**
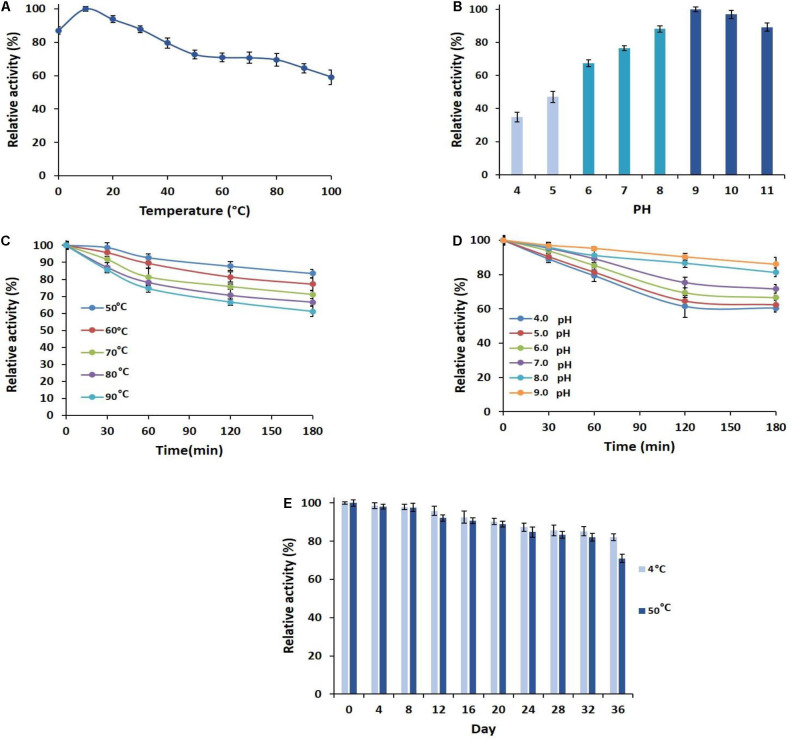
Characterization of PersiAmy1. **(A)** Effect of temperature on the activity of PersiAmy1. **(B)** Effect of pH on the activity of PersiAmy1 using carbonate-bicarbonate buffer (pH 4.0–5.0), phosphate buffer (pH 6.0–8.0), and Tris–HCL buffer (pH 9.0–11.0). **(C)** Thermostability of PersiAmy1 on various temperatures (50–90°C). **(D)** pH stability of PersiAmy1 after a 180-min incubation at different pH using carbonate-bicarbonate buffer (pH 4.0–5.0), phosphate buffer (pH 6.0–8.0), and Tris–HCL buffer (pH 9.0). **(E)** Storage stability of PersiAmy1 after 36 days at 4 and 50°C.

To investigate the optimum temperature of the enzyme, its activities in various temperatures from 0 to 100°C were measured (Figure 2A). The greatest relative activity at 10°C indicated the optimum temperature and the cold adaptation of PersiAmy1. PersiAmy1 retained nearly 60% of its maximum activity at 100°C and showed 88% activity at 0°C which confirmed its activity at a broad range of temperatures. The effect of pH on the activity of PersiAmy1 was tested at a pH range from 4 to 11 at the optimum temperature (Figure 2B). The enzyme was active at all of the assayed pH values and reached the highest activity at pH 9.0 which is the optimum value and supported the alkali tolerance of the enzyme.

The heat sensitivity of the PersiAmy1 was analyzed at 50–90°C, and the results are illustrated in Figure 2C. PersiAmy1 was stable after 180 min of incubation at various temperatures. The purified α-amylase demonstrated 83.50, 77.16, 71.03, 66.60, and 61.09% activities after 180 min at 50, 60, 70, 80, and 90°C, respectively. Based on the results of pH stability in Figure 2D, it is observed that PersiAmy1 is stable at alkaline conditions and is active under acidic situations. α-Amylase retained 60.48, 62.38, 66.52, 71.64, 81.26, and 86.05% activities after 180 min at pH 4.0, pH 5.0, pH 6.0, pH 7.0, pH 8.0, and pH 9.0, respectively, which intended the broad range of activity and stability for PersiAmy1.

### Substrate Spectrum of the Purified PersiAmy1

According to the substrate spectrum of PersiAmy1, it is observed that the purified enzyme had a high ability to degrade various polysaccharide substrates (Table 1). The highest activity was found by amylopectin (100% relative activity and 7515.95 U.mg^–1^ specific activity) and the lowest activity obtained in the presence of glycogen (42.55% relative activity and 3198.73 U.mg^–1^ specific activity). PersiAmy1 not only showed the capacity to hydrolyze the substrates with both α-1,4 and α-1,6 linkages but also degraded the amylose with the main linkage of α-1,4, and pullulan with the major bond of α-1,6 with 50.76 and 61.70% relative activities, respectively.

**TABLE 1:** Substrate specificity of the purified PersiAmy1.

Substrate	Main linkage	Relative activity (%)	Specific activity (U/mg)
Amylopectin	α-1,4 and α-1,6	100	7515.98
Amylose	α-1,4	50.76	3815.48
Glycogen	α-1,4 and α-1,6	42.55	3198.73
Pullulan	α-1,6	61.70	4637.81
Wheat starch	α-1,4 and α-1,6	54.05	4062.88
Potato starch	α-1,4 and α-1,6	51.41	3864.26
Corn starch	α-1,4 and α-1,6	61.98	4658.72

### Kinetic Studies of the Enzyme Reaction

The kinetic parameters of PersiAmy1 were determined by using the starch as a substrate. Results of this study confirmed the matching of the PersiAmy1 properties with the Michaelis–Menten kinetics. The K_m_ and V_max_ values of the purified enzyme were 0.37 mg.ml^–1^ and 74.08 μmol.ml^–1^.min^–1^, respectively.

### Storage Stability of the Robust Stable PersiAmy1

The effect of the long-term storage on the activity and stability of PersiAmy1 was determined by incubating the enzyme for 36 days at two different temperatures (4 and 50°C). As illustrated in Figure 2E, the enzyme showed strong stability under both temperatures after 36 days of storage. The enzymatic activity was higher at 4°C compared with 50°C, and the purified α-amylase retained 82.13 and 71.01% of its maximum activity at 4 and 50°C after 36 days, respectively.

### Evaluation of the Robust Stable PersiAmy1 Stability in the Presence of Metal Ions and Inhibitors

**FIGURE 3:**
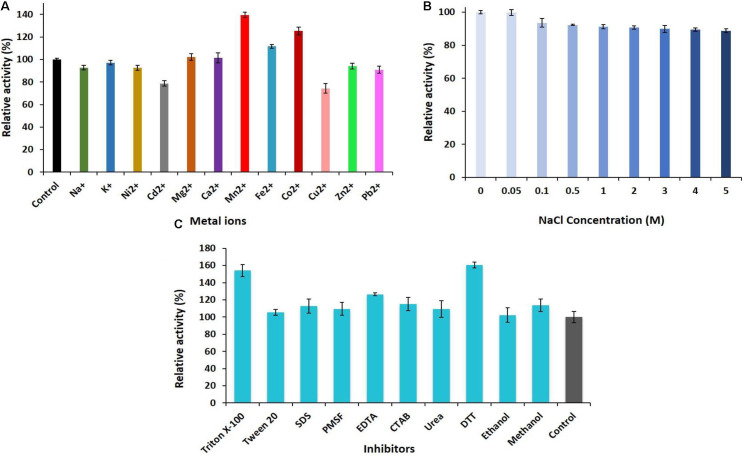
**(A)** Effect of metal ions. **(B)** Various concentrations of NaCl (0–5 M). **(C)** Different concentrations of the inhibitors and surfactants on the activity of PersiAmy1.

The ability of PersiAmy1 to withstand multivalent metal ions was investigated (Figure 3A). The enzyme was stable at the presence of different metal ions, and Mn^2+^, Co^2+^, and Fe^2+^ enhanced the enzymatic activity to 139.54, 125.25, and 111.59%, respectively. The slight inhibition was found by Cd^2+^ and Cu^2+^ with the relative activities of 78.67 and 74.32%.

The halotolerance of PersiAmy1 was analyzed at different concentrations of NaCl (Figure 3B). The purified enzyme was highly stable at high concentrations of NaCl as well as low concentrations. α-Amylase displayed 99.79% activity at 0.05 M NaCl and reached 88.81% activity in the presence of 5 M NaCl.

The effect of various inhibitors and surfactants in 10 mM concentrations on the activity of the PersiAmy1 was explored (Figure 3C). Based on the experimental results, PersiAmy1 was active and stable in the presence of all additives. PersiAmy1 showed remarkable resistance different inhibitors including Triton X-100, Tween 20, SDS, PMSF, EDTA, CTAB, urea, DTT, ethanol, and methanol, and addition of these inhibitors at the concentration of 10 mM did not affect the amylase activity.

### Potential Application of the Robust Stable PersiAmy1 in Detergent Industry

#### Compatibility With Commercial Detergents

**FIGURE 4:**
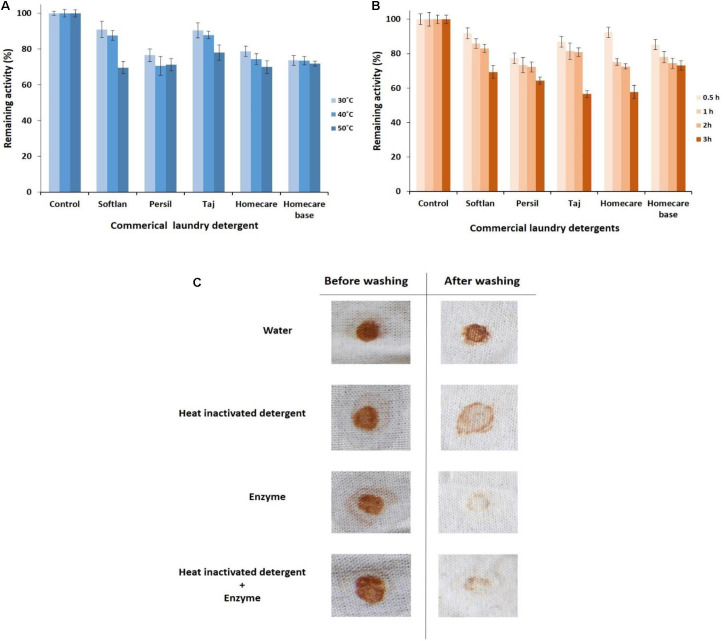
Detergent compatibility study of the purified PersiAmy1. **(A)** Remaining activities after incubating in various temperatures (30, 40, and 50°C). **(B)** Remaining activities after incubating in various times (0.5, 1, 2, and 3 h). **(C)** Wash performance analysis of the purified PersiAmy1.

The detergent compatibility of PersiAmy1 was also tested by incubating the enzyme and detergents at different temperatures. Results showed that the enzyme retained more activity at 30°C than at 40 and 50°C (Figure 4A). Interestingly, the alkaline cold-adapted PersiAmy1 was extremely stable toward all detergents tested and showed more than 73% enzymatic activity at 30 and 40°C and more than 69% activity at 50°C.

To study the compatibility of PersiAmy1 with commercial detergents, the enzyme was preincubated in the presence of several laundry detergents at various times (0.5–3 h) and temperatures (30–50°C). As shown in Figure 4B, the most remaining activity of the enzyme was obtained after 0.5 h at its optimal temperature (10°C) and decreased by enhancing the incubation time. The stable PersiAmy1 retained more than 70 and 50% of its activity after 2 and 3 h of preincubation with all the tested detergents, respectively. The maximum remaining activity of the enzyme was observed in the presence of Softlan, Persil, and Homecare base (more than 60%) and lower activity gained by Taj and Homecare (more than 50%) after 3 h.

#### Wash Performance Analysis

The stain removal ability of the purified alkaline α-amylase was assessed by using chocolate-stained cotton fabrics. Stained cotton fabrics were washed by four different sets of washing solutions. The best destaining was observed in the presence of detergent supplemented with the purified alkaline α-amylase, as evident in Figure 4C. There was considerable loss of stain with PersiAmy1 alone, which supports the hypothesis that the enzyme could be used as a detergent additive.

### Potential Application of the Robust Stable PersiAmy1 in Bakery Industry

The effect of PersiAmy1 on the textural properties and color of the wheat bread was investigated, and the results are illustrated in Table 2. Significant differences were obtained in the hardness, springiness, chewiness, and cohesiveness of the samples (*p* < 0.05). Hardness of the bread decreased from 10.19 to 5.88 N in the presence of PersiAmy1 and showed a softer texture in crumbs. Also, the addition of the enzyme increased the springiness of the bread and showed values of 10.44 N and 11.74 N for the control and α-amylase treated bread, respectively. The cohesiveness and chewiness of the bread decreased from 0.46 and 53.30 mJ in the control sample to 0.40 and 31.06 mJ in enzyme-treated bread, respectively.

**TABLE 2:** Texture and color analysis of wheat bread before and after addition of enzyme.

Texture analysis				

Sample	Hardness (N)	Springiness (mm)	Cohesiveness	Chewiness (mJ)
**Control**	10.19 ± 2.51	10.44 ± 0.13	0.46 ± 0.04	53.30 ± 11.34
**α-Amylase treated**	5.88 ± 1.03	11.74 ± 0.27	0.40 ± 0.02	31.06 ± 5.29
***F* value**	7.50*	54.88*	8.80*	32.91*

**Color analysis**				

**Sample**	L*	a*	b*	**BI**

**Control**	87.75 ± 1.48	−2.81 ± 2.74	10.75 ± 2.35	10.41 ± 4.23
**α-Amylase treated**	83.48 ± 1.41	−0.45 ± 0.31	13.44 ± 2.65	16.82 ± 3.71
***F* value**	43.30*	7.25*	5.77*	12.91*

*Differences tested on the 0.95 level (p < 0.05).*

The color analysis showed that the brightness (L^∗^) of the samples was decreased (*p* < 0.05) from 87.75 to 83.48, which proposed the darker crust color after the addition of PersiAmy1 (Table 2). The a^∗^ of control bread increased after the addition of amylase and showed the tendency to redness. Moreover, the yellowness (b^∗^) of the bread was enhanced in the presence of enzyme. The browning index of samples was also increased after the addition of PersiAmy1 and developed from 10.41 to 16.82. Enhancement of the browning index of bread can be due to the increase in the amounts of released reducing sugar at the presence of α-amylase which affected the yeast function and improved the fermentation condition and Maillard reaction (Goesaert et al., 2009).

**FIGURE 5:**
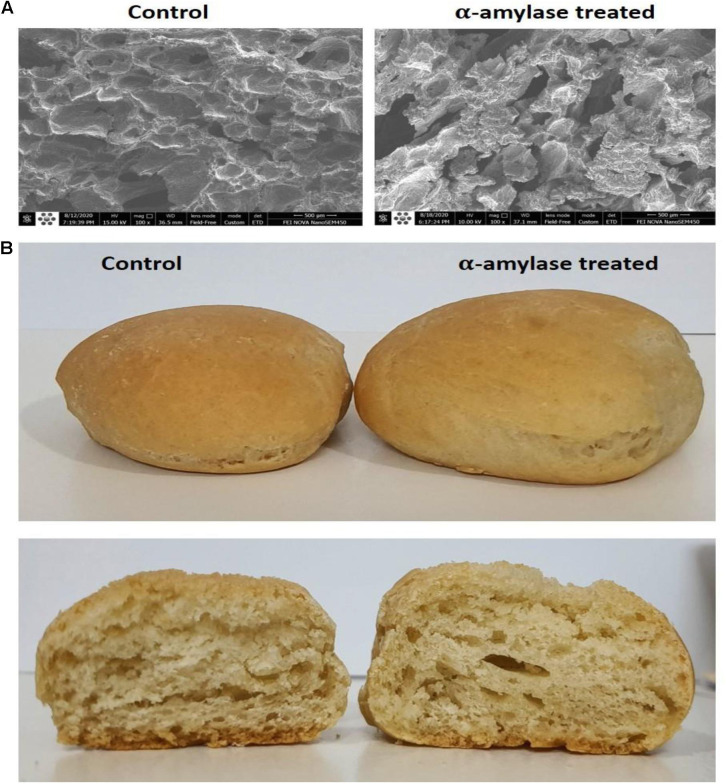
Images of the treated wheat breads. **(A)** Scanning electron micrographs under a magnification of 100×. **(B)** Crumb and crust of the wheat bread samples before and after addition of PersiAmy1.

As shown in Figure 5, PersiAmy1 positively affected the porosity of the crumb and produced larger holes compared with the control sample. This is because of the increase in production of reducing sugar, gas production, and creation of the bubbled cell structure. Moreover, the addition of PersiAmy1 correlated to an increased bread volume and improved the quality of the final product.

## Discussion

Nowadays, there is an urgent demand for highly stable α-amylases to meet the industrial applications. Therefore, it is inevitable to search for novel α-amylases that could withstand harsh industrial process conditions. Because metagenomic data can be a great source for identifying novel enzymes with valuable properties in the industry, sheep metagenomic data were used in this study. Nevertheless, given the value of this data in discovering new enzymes, choosing the best enzyme is the challenge. In this study, the sheep rumen metagenome was explored using computational screening for finding a novel robust stable α-amylase. The main advantage of applying our proposed computational method is that instead of simple alignment-based screening, a multistep procedure is employed to refine a vast number of potential α-amylases and narrow down the list of candidates to a minimal number of enzymes with superior desired properties. In this methodology, the relatively high cost of functional screening is replaced by computational costs.

Biochemical characterization of the alkaline cold-adapted PersiAmy1 showed that the enzyme was strongly active under downstream conditions, which is essential for using the enzyme in several industrial processes. PersiAmy1 withstood extreme pH and temperature and was active and stable at wide temperature and pH ranges. Comparison of the PersiAmy1 properties with reported cold-active and/or alkaline α-amylases indicated the high stability of the enzyme. Possessing a powerful function in the detergent industry, cold active alkaline α-amylases have been intensely studied. A cold adapted α-amylase from metagenomic library showed optimum pH of 8–9 and temperature of 10–15°C (Vester et al., 2015). In other studies, a cold-adapted and salt-tolerant α-amylase from *Pseudoalteromonas* and alkaline α-amylase from *Bacillus mojavensis* showed optimum reaction condition at 25°C, pH 8 and 55°C, pH 9, respectively (Hammami et al., 2018; Wang et al., 2018). Thermostability, pH tolerance, and cold activity are the desired characteristics for α-amylase in bakery and detergent industries. According to Table 3, among the different α-amylases, PersiAmy1 demonstrated the highest activity at 0°C. The low temperature stability is so crucial for α-amylase to be used as an additive in detergent where it allows eco-friendly, low-temperature washing and considerable progress toward energy savings (Al-ghanayem, 2020). Moreover, both the thermal and pH stabilities of PersiAmy1 were retained to a considerable level after 180 min of incubation, showing 86% activity after 180 min at pH 9.0 and 61% activity after 180 min at 90°C. α-Amylases with thermo-alkali stability are the premier choices as bio-additives in the detergent and bakery industry. As a detergent additive, the alkaline α-amylases are promising candidates due to the high pH of detergents and their effect on reducing damage to the textile fabric. Moreover, supplementation of bread with thermostable α-amylase delays breadcrumb firming and has a relevant impact on the texture and sensory properties of bread. The high stability of PersiAmy1 under broad temperature and pH ranges are desired properties for wide industrial applications.

**TABLE 3:** Property comparison of PersiAmy1 with some known cold-active and/or alkaline α-amylases.

Source	pH stability	Thermal stability	Cold activity	References
*Bacillus* SP-CH7	>80% activity after 60 min at pH 13.0	<20% activity after 60 min at 95°C	<20% activity at 15°C	Priyadarshini and Ray, 2019
*Bacillus subtilis* N8	50% activity after 24 h at pH 12.0	83% activity after 60 min at 40°C	91% activity at 10°C	Arabacı and Arıkan, 2018
*Pseudoalteromonas* M175	<80% activity after 60 min at pH 9.0	<10% activity after 60 min at 50°C	<50% activity at 0°C	Wang et al., 2018
*Bacillus mojavensis*	<70% activity after 60 min at pH 9.0	<40% activity after 60 min at 70°C	40% activity at 30°C	Hammami et al., 2018
*Geomyces pannorum*	80% activity after 30 min at pH 9.0	0% activity after 15 min at 60°C	<30% activity at 0°C	He et al., 2017
*Exiguobacterium* SH3	–	<10% activity after 30 min at 60°C	41% activity at 0°C	Emampour et al., 2015
*Arthrobacter agilis*	<60% activity after 60 min at pH 9.0	>80% activity after 60 min at 70°C	–	Kim et al., 2017
*Bacillus methylotrophicus*	<100% activity after 60 min at pH 9.0	<60% activity after 60 min at 50°C	<20% activity at 20°C	Hmidet et al., 2019
*Bacillus pseudofirmus*	80% activity after 12 h at pH 9.0	<80% activity after 20 min at 50°C	40% activity at 30°C	Lu et al., 2016
Metagenome-derived	<80% activity after 30 min at pH 9.0	<10% activity after 30 min at 90°C	<20% activity at 2°C	Nair et al., 2017
Metagenome-derived	>80% activity after 14 h at pH 9.0	<10% activity after 60 min at 50°C	70% activity at 1°C	Vester et al., 2015
Metagenome-derived (PersiAmy1)	86% activity after 180 min at pH 9.0	61% activity after 180 min at 90°C	87% activity at 0°C	This study

The high activity of α-amylases toward a broad range of substrates is an important phenomenon due to the key role of these enzymes in many industrial processes (Fang et al., 2019). The thermostable nature of the α-amylases coupled with acting on a wide array of substrates is an attractive feature suitable for specific applications such as the bakery industry (Verma et al., 2020). In this study, results from substrate specificity analysis revealed the ability of PersiAmy1 to break both the α-1,4 and α-1,6 linkages of the polysaccharide substrates. Previous cold-active α-amylases showed the activity on different starches as well as amylose and amylopectin (Qin et al., 2014; Emampour et al., 2015), but they did not show the activity on pullulan with α-1,6 linkages which signified the broad range of substrate activity of PersiAmy1 compared with other known cold-adapted α-amylases.

As K_m_ is considered as the affinity of the enzyme toward substrate, the lower value of K_m_ represents the higher initial rate of the reaction and affinity of enzyme for substrate. The K_m_ value of PersiAmy1 was lower than the cold-active α-amylases from *Zunongwangia profunda*, *Pseudoalteromonas* sp. M175, *Exiguobacterium* (Qin et al., 2014; Emampour et al., 2015; Wang et al., 2018), and a cold, alkaline, metagenome-derived α-amylase (Vester et al., 2015). Based on the results of kinetic analysis, PersiAmy1 showed higher V_max_ than detergent-stable alkaline α-amylase from *Bacillus* showing V_max_ of 2.342 μmol.ml^–1^.min^–1^ (Priyadarshini and Ray, 2019). Moreover, another cold-adapted α-amylase from *Monascus sanguineus* and an alkaline α-amylase from *Alkalimonas amylolytica* showed V_max_ of 22.07 μmol.ml^–1^.min^–1^ and 37.8 μmol.ml^–1^.min^–1^, respectively, when starch was used as substrate (Yang et al., 2013; Tallapragada et al., 2017).

Improving the shelf-life and stability of the enzyme is the critical point in industrial operations, and several techniques have been developed for increasing the stability of the enzyme (Advances, 2018). Production of the highly stable enzyme can be a cost-effective way to increase the enzyme efficiency without applying costly and time-consuming methods. The storage stability of the purified enzyme implied the superior ability of PersiAmy1 to maintain its stability in long-term storage which is the major concern in most industrial processes. Despite the high stability of the cold-adapted enzyme at low temperature (4°C), α-amylase showed strong stability at a higher temperature (50°C).

In addition to considerable temperature and pH stability, PersiAmy1 was stable in the presence of different inhibitors, detergents, and metal ions. In this study, the PersiAmy1 activity was estimated against different metal ions and maximum enzyme activity found by Mn^2+^ and Co^2+^. This result agrees with amylase from *Aureobasidium pullulans* and *Pseudomonas balearica* activated by Co^2+^ and Mn^2+^, respectively (Mulay and Deopurkar, 2018; Kizhakedathil and Subathra Devi, 2021). Generally, some metal ions are involved in enhancing the α-amylase activity and acting as an activator and accelerator for the enzyme. The stimulated activity of PersiAmy1 in the presence of Mn^2+^ and Co^2+^ might be due to the role of these ions in maintaining the α-amylase structure, catalytic activity, and stability. These ions may also be involved in the relationship between the enzyme and the substrate, the stabilizing charges, and the transfer state stabilization (Prakash and Jaiswal, 2010; Adefila et al., 2012). The enzyme activity was also dropped to 74.32 and 78.67% when α-amylase was incubated with Cu^2+^ and Cd^2+^ ions. This result was higher than the activity of thermostable and alkaline α-amylase from *Tepidimonas fonticaldi* and thermostable α-amylase from *Trichoderma pseudokoningii* in the presence of 5 mM Cu^2+^ and Cd^2+^ (Abdulaal, 2018; Allala et al., 2019).

Most of the α-amylases rely on divalent ions, especially Ca^2+^ for their activity maintenance, and EDTA partially or completely inhibits the amylolytic activity (Gupta et al., 2003). The slight increase of the PersiAmy1 activity in the presence of EDTA indicated that this inhibitor might not be acting as a chelating agent for PersiAmy1 which is consistent with previous studies (Allala et al., 2019; Priyadarshini and Ray, 2019). EDTA is a common efficient chelator of enzyme cofactors, and a slight increase in α-amylase activity due to EDTA shows that this compound may have had a positive influence on enzymatic activity which related to the structure–function relationship of the enzyme (Naika and Tiku, 2011). Different inhibitors such as EDTA, PMSF, and DTT have been introduced as strong inhibitors for α-amylase activity (Mehta and Satyanarayana, 2016). PersiAmy1 was not affected by two inhibitors of α-amylase activity, DTT and PMSF, which act on the -SH groups and the seryl hydroxyl group of the enzyme (Mehta and Satyanarayana, 2016). The reason behind the increased activity of PersiAmy1 in the presence of DTT might be due to the protection of the enzymes –SH groups by thiol reagent and, as a result, their consumption for the catalytic activity. Furthermore, the enzyme was stable in the presence of Tween 20, CTAB, urea, SDS, and organic solvents such as ethanol and methanol. Presence of the non-ionic surfactant, Triton X-100, increased the enzymatic activity up to 154.18% suggesting the better electrostatic stability in solution reaction after interaction of the surfactant and enzyme.

Moreover, PersiAmy1 has wide-range salt tolerance and retains high activity (88.81%) at high NaCl concentration (5 M). This result was higher than a halotolerant recombinant α-amylase from halophilic archaeal strain and halotolerant α-amylase from the marine metagenomic library (Nair et al., 2017; Verma et al., 2020). Indeed, the halotolerant, detergent-compatible PersiAmy1 could be used as a suitable alternative in the detergent industry since it presented good stabilities at different factors essential for de-staining.

Detergents are essential cleaning products with a considerable number of industrial applications. In laundry operations, the preference is to use cold water for washing to avoid damage to fabrics. Most of the α-amylases inactivate easily by the harmful chemicals in detergents; thus, the demand for α-amylases with desired properties for detergent industry has increased (Al-ghanayem, 2020). The enzyme must be alkali and detergent stable for the application as a detergent additive. PersiAmy1 showed high remaining activity when incubated with commercial detergents at a different time and at 10°C. Moreover, the considerable stability of α-amylase after the supplementation with commercial detergents at different temperatures (30–50°C) showed its efficiency in the detergent industry. The activity of PersiAmy1 in the presence of commercial detergents (7 mg/ml) at different temperatures (30–50°C) was higher than previous alkaline cold-active α-amylases (Hammami et al., 2018; Hmidet et al., 2019). Also, the enzyme showed more than 73% activity after incubation with all the commercial detergents for 1 h at 10°C, which is higher than α-amylase from *Pseudoalteromonas*, which showed 76% activity at 25°C (Wang et al., 2018). The high stability of PersiAmy1 versus detergent components and its suitable stain removal ability are evident for the potential of PersiAmy1 as a detergent additive.

The addition of the PersiAmy1 bread formulation reflected the efficiency of amylase in the food industry. The enzyme produced a softer and more chewable loaf and significantly decreased the hardness and chewiness of the bread. The same results were reported in the previous study using the new thermostable α-amylase from *Rhizomucor miehei* (Wang et al., 2020). Another study reported an improved volume and structure of the bread using a novel cold-adapted amylase (He et al., 2017). Moreover, PersiAmy1 showed better efficiency on bread with a lower concentration of enzyme (0.008 U/g) than α-amylase from *Bacillus subtilis* which added to the bread in 0.04 and 0.06 U/g concentrations (Trabelsi et al., 2019). These outcomes implied that the strong activity and stability of PersiAmy1 provided a great opportunity for using the enzyme in the food industry and bakery products.

In summary, using a cost-effective multistage screening pipeline resulted in identifying the highly stable PersiAmy1 from the rumen metagenomic data. PersiAmy1 showed long-term stability against chemical inhibitors, detergents, extreme temperature, and pH as well as extension. The suburb performance of PersiAmy1 represented the enzyme potency in the detergent formulation and bakery industry. The compatibility of the enzyme with commercial detergents and effective destaining of fabric supported its candidature in the detergent industry, even at low temperatures. Since the enzyme effectively reduces the bread hardness and increases the porosity and volume of the bread, it can find application in the bakery industry. The robust, stable PersiAmy1 is suggested as a potential α-amylase for a wide array of industrial processes by focusing on high activity and stability under denaturing conditions.

## Data Availability Statement

The original contributions presented in the study are included in the article/Supplementary Material, further inquiries can be directed to the corresponding author/s.

## Author Contributions

SA: conceptualization, methodology, supervision, investigation, writing – original draft, resources, and project administration. BZ: help to data analysis, review, and editing. SS: methodology, analysis and interpretation the data, and writing – original draft. KK: conceptualization, methodology, contribution to the computational and bioinformatics data analysis, and writing – review and editing. MM: performing the gene cloning, expression, and purification. SR: performing and checking data analysis. GH: supervision, resources, project administration, funding acquisition, and conceptualization. All authors contributed to the article and approved the submitted version.

## Conflict of Interest

The authors declare that the research was conducted in the absence of any commercial or financial relationships that could be construed as a potential conflict of interest.

## Publisher’s Note

All claims expressed in this article are solely those of the authors and do not necessarily represent those of their affiliated organizations, or those of the publisher, the editors and the reviewers. Any product that may be evaluated in this article, or claim that may be made by its manufacturer, is not guaranteed or endorsed by the publisher.
