# Supplementary material for: Highly Efficient Computationally Derived Novel Metagenome α-Amylase With Robust Stability Under Extreme Denaturing Conditions
Source: Front Microbiol. 2021 Aug 30;12:713125. doi: 10.3389/fmicb.2021.713125 (PMC8437397; doi:10.3389/fmicb.2021.713125)
Supplement: Supplementary file 1 [file Data_Sheet_1.PDF]

Highly efficient computationally derived novel metagenome α-amylase with robust stability under extreme denaturing conditions

Shohreh Ariaeenejad[a]*, Behrouz Zolfaghari[b], Seyedeh Fatemeh Sadeghian Motahar[a], Kaveh Kavousi[c], Morteza Maleki[a], Swapnoneel Roy[d], Ghasem Hosseini Salekdeh[a,e]*

[a] Department of Systems and Synthetic Biology, Agricultural Biotechnology Research Institute of Iran (ABRII), Agricultural Research Education and Extension Organization (AREEO), Karaj, Iran.

[b] CSE Department, Indian Institute of Technology (IIT) Guwahati, Guwahati, Assam, India.

[c] Laboratory of Complex Biological Systems and Bioinformatics (CBB), Department of Bioinformatics, Institute of Biochemistry and Biophysics (IBB), University of Tehran, Tehran, Iran.

[d] School of Computing, University of North Florida, Jacksonville, FL, USA.

[e] Department of Molecular Sciences, Macquarie University, Sydney, NSW Australia.

* Corresponding authors:

Shohreh Ariaeenejad (shariaee@gmail.com; sh.ariaee@abrii.ac.ir)

And

Ghasem Hosseini Salekdeh (h_salekdeh@abrii.ac.ir; hsalekdeh@yahoo.com)

Department of Systems and Synthetic Biology, Agricultural Biotechnology Research Institute of Iran (ABRII), Karaj, Iran.

P. O. Box: 31535-1897, Tel.: +98 26 32703536, Fax: +98 26 32704539,

Supplementary material

Table.1- Some alkaline or/and cold-stable α-amylase mined from the literature and used for standalone NCBI BLAST.

Row	Gen bank	Properties	Ref
1	ANR02572.1	38% residual activity at 10 ◦C<br>Optimally active at 40 ℃, pH6.2	(Song et al., 2016)
2	AKX30796.1	Cold-active α-amylase	(Souter, et al, 2012)
3	AGO71121.1	Cold-active detergent-stable α-amylase	(Amin, Neelam et al, 2013)
4	PHS15093.1	Cold-active α-amylase	(Tully et al., 2018)
5	ALE05211.1	Cold-active α-amylase	(Kumar et al., 2015)
6	KHL92445.1	Cold-active α-amylase	(Dhar et al., 2015)
7	AMB59661.1	Cold-active α-amylase	(Swarnkar et al., 2016)
8	AQM89788.1	Cold-active α-amylase	(Bessler, et al 2016)
9	ACW41737.1	Cold-active α-amylase	(Kasturi, et al, 2009)

Figure 1. Sequence data of PersiAmy1 have been deposited in the GenBank database under accession number (MT560082).

LOCUS BSeq#1 1434 bp DNA linear ENV 03-JUN-2020

DEFINITION Uncultured bacterium plastid clone recombinant plasmid pet28-PersiAmy1. map Iran.

SOURCE plastid uncultured bacterium

ORGANISM uncultured bacterium

Bacteria; environmental samples.

REFERENCE 1 (bases 1 to 1434)

AUTHORS Ariaeenejad,S. and Hosseini Salekdeh,G.

TITLE PersiAmy1

source 1..1434

/organism="uncultured bacterium"

/organelle="plastid"

/mol_type="genomic DNA"

/isolation_source="Sheep rumen"

/db_xref="taxon:77133"

/map="Iran"

/clone="recombinant plasmid pet28-PersiAmy1."

/country="Iran"

/collection_date="2018"

/collection_date="20-Feb-2018"

/note="[uncultured (using universal primers)]"

CDS 1..1434

/note="Alpha Amylase from sheep rumen uncultured"

/codon_start=1

/transl_table=11

/product="PersiAmy1"

/translation="MKKYLLPILCLLAVACGGNGKTALTSIPAPEDVVMYQINPRNFA

PDHSFQAVSARLDAIQELGANVVWFMPICEIGVEKAVQSPYCVKNYTAVNPEFGTLED

FKALVDGAHQRGMAVIIDWVANHTSWDNPWIKDHPEWYTHNEAGDIISPAGTGWNDVA

DLNFDNPDLCQAMIDAMKFWVEEVGVDGFRCDAADYVPFEFWKDCVAQLRATGHELLM

LAEGQRKDHFDADFDMNYAWGWLSALRRVYTGVTETIQQPVPQRQQGNRQGQNARQGN

RPQGQQPQMRTRTVNRAVPVSTLFASDSSEYAGLPAGRVKLRFTTNHDEHVKNSPVRE

FFGNDGSVAAFVATTFIHGGMLIYGCQEVGYPGKINFFRYAEIDWDANPDMFNAYKQI

VRVFKETPAIRRGALVPYPHNDILIFERVLDNEKVLVMVNMRDTQLEAPVPAAWQGRT

ATDLITRKSVSFGETERLRPFEYVIVK"

BASE COUNT 295 a 504 c 379 g 256 t

ORIGIN

1 atgaaaaagt acctcttacc cattctttgc ctgctcgccg tggcctgcgg cggcaacggg

61 aaaacagcgc tgacgtccat cccggctccg gaagatgtcg tcatgtatca gatcaatccg

121 cgcaactttg caccggacca ttctttccag gccgtttccg cccgtctgga cgccatccag

181 gaactgggcg ccaacgtcgt ctggttcatg ccaatctgcg aaatcggcgt agagaaagcc

241 gtccagagcc cctattgcgt caagaactat accgccgtca accctgaatt cggcaccctc

301 gaggacttca aggccctcgt ggacggcgcc caccagcgcg gcatggccgt catcatcgac

361 tgggtcgcca accacacttc ctgggacaat ccgtggatca aggaccatcc ggaatggtac

421 acccacaacg aagccggcga catcatcagt ccggccggta ccggctggaa cgatgtggcg

481 gacctgaact tcgacaaccc ggatctctgc caggcgatga tcgacgccat gaaattctgg

541 gtcgaggagg tcggtgtcga cggtttccgc tgcgacgccg ccgactatgt cccgtttgaa

601 ttctggaagg actgcgtcgc ccagctccgc gctaccggcc atgaactcct catgctcgcc

661 gaaggccagc gcaaggacca tttcgacgcc gactttgaca tgaactacgc atggggctgg

721 ctttccgccc tccgtcgcgt ctacaccggc gtgaccgaaa ccatccagca gccggtgccg

781 cagcgtcagc aaggcaaccg ccagggccag aacgcccgcc agggcaatcg cccgcagggc

841 cagcagccgc agatgcgtac ccgcaccgtc aaccgggccg ttccggtctc caccctcttc

901 gcctccgact ccagcgagta tgccggcctg ccggccggac gcgtcaagct ccgtttcacg

961 accaaccatg acgaacatgt caagaactcc ccggtccgtg aattcttcgg caacgacggt

1021 tccgtcgccg cgttcgtcgc caccaccttc atccacggcg gcatgctgat ctacggatgc

1081 caggaagtcg gctatccggg caagatcaat ttcttccgct atgccgaaat cgactgggac

1141 gccaatcccg acatgttcaa cgcctacaag cagatcgtcc gcgtcttcaa ggagaccccg

1201 gccatccgcc gcggcgccct cgtcccgtat ccgcacaacg acatcctcat ctttgaacgc

1261 gtcctggaca acgagaaggt cctcgtgatg gtcaacatgc gcgacaccca gctcgaagcc

1321 ccggttcccg ccgcatggca gggacgcacg gcgaccgacc tgatcacccg caagtccgtc

1381 tccttcgggg agaccgaacg gctccgtcct ttcgaatacg tcatcgtcaa ataa

Figure 2. The phylogenetic tree constructed with Neighbor Joining method. From an evolutionary perspective, the PersiAmy1 is most similar to cold active alkalophilic alpha amylase.

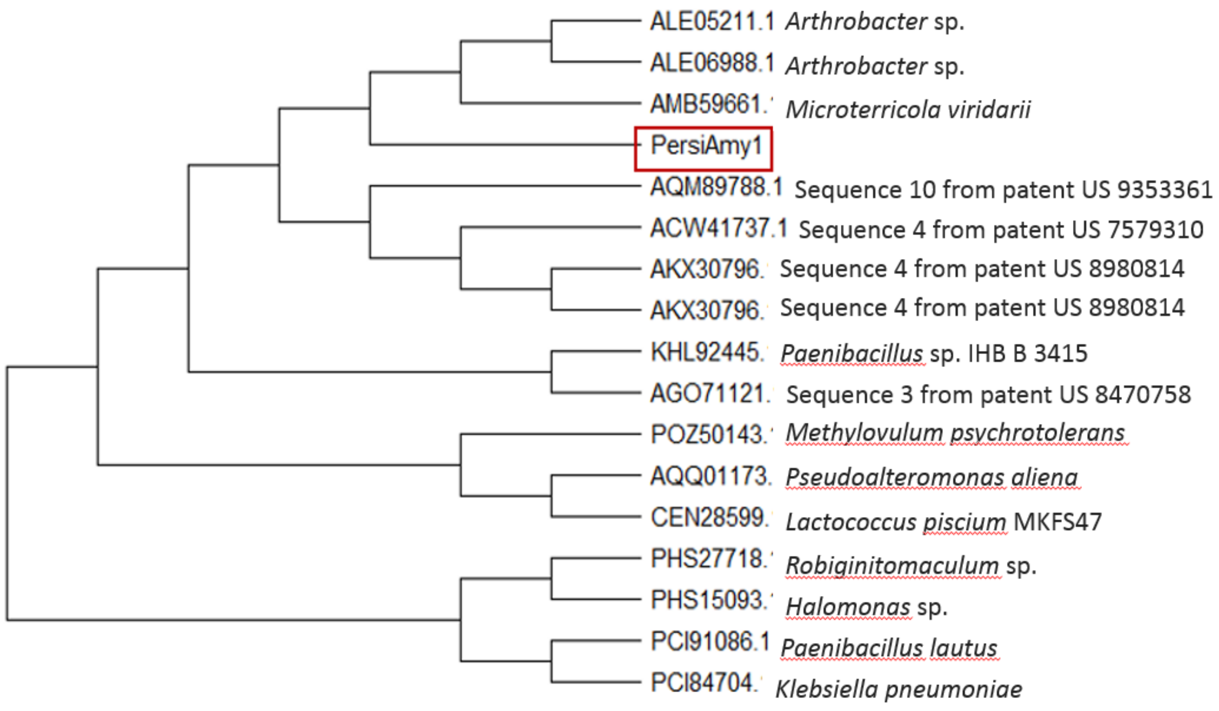

References

Amin, Neelam S and Estabrook, Melodie and Jones, Brian E and Kolkman, Marc and Vroemen, Casper and Weyler, W., 2013. Detergent compositions and methods of use for an alpha-amylase polypeptide of bacillus species 195. Google Patents.

Bessler, Cornelius and Wieland, Susanne and Maurer, K.-H., 2016. Alpha-amylase variants stabilized against dimerization and/or multimerization, method for the production thereof, and detergents and cleansers containing these alpha-amylase variants. Google Patents.

Dhar, H., Swarnkar, K., Gulati, A., Singh, K., Kasana, C., 2015. Draft Genome Sequence of a Cellulase-Producing Psychrotrophic Paenibacillus Strain , IHB B 3415 , Isolated from the Cold Environment of the Western Himalayas , India 3, 1–2. https://doi.org/10.1128/genomeA.01581-14.Copyright

Kasturi, Chandrika and Wandstrat, Mark Edward and Song, B.X., 2009. Liquid detergent composition exhibiting enhanced α-amylase enzyme stability. Google Patents.

Kumar, R., Singh, D., Swarnkar, M.K., Singh, A.K., Kumar, S., 2015. Complete genome sequence of Arthrobacter sp. ERGS1:01, a putative novel bacterium with prospective cold active industrial enzymes, isolated from East Rathong glacier in India. Elsevier B.V. https://doi.org/10.1016/j.jbiotec.2015.09.025

Song, Q., Wang, Y., Yin, C., Zhang, X., 2016. Enzyme and Microbial Technology LaaA , a novel high-active alkalophilic alpha-amylase from deep-sea bacterium Luteimonas abyssi XH031 T. Enzyme Microb. Technol. 90, 83–92. https://doi.org/10.1016/j.enzmictec.2016.05.003

Souter, Philip Frank and Ward, Glenn Steven and Goedegebuur, Frits and Poulose, Ayrookaran Joseph and Estell, David A and Bott, Richard R and Casc{\"a}o-Pereira, L.G., 2012. Automatic Dishwashing Detergent Composition. Google Patents.

Swarnkar, M.K., Singh, D., Kumar, R., 2016. First complete genome sequence of a species in the genus Microterricola , an extremophilic cold active enzyme producing bacterial strain ERGS5 : 02 isolated from Sikkim Himalaya. J. Biotechnol. 222, 17–18. https://doi.org/10.1016/j.jbiotec.2016.02.011

Tully, B.J., Wheat, C.G., Glazer, B.T., Huber, J.A., 2018. ORIGINAL ARTICLE A dynamic microbial community with high functional redundancy inhabits the cold , oxic subseafloor aquifer 1–16. https://doi.org/10.1038/ismej.2017.187
